# The impact of height and weight on rescreening rates within a population‐based breast screening program

**DOI:** 10.1002/cam4.6883

**Published:** 2024-01-11

**Authors:** Sarah Pirikahu, Ellie Darcey, Helen Lund, Elizabeth Wylie, Jennifer Stone

**Affiliations:** ^1^ Genetic Epidemiology Group, School of Population and Global Health University of Western Australia Perth Western Australia Australia; ^2^ BreastScreen Western Australia Women and Newborn Health Service Perth Western Australia Australia; ^3^ Medical School University of Western Australia Perth Western Australia Australia

**Keywords:** breast cancer, obesity, participation, risk factor, screening

## Abstract

**Introduction:**

Women with obesity are at increased risk of post‐menopausal breast cancer and less likely to participate in breast screening. This study investigates the impact of asking women their height and weight within a population‐based screening program, and the association of BMI with rescreening status.

**Methods:**

Data regarding 666,130 screening events from 318,198 women aged 50–74 attending BreastScreen Western Australia between 2016 and 2021 were used to compare crude and age‐standardised rescreening rates over time. Mixed effects logistic regression was used to investigate associations of BMI with rescreening status.

**Results:**

Rescreening rates for women screened since 2016 were within 1.8% points from the previous reporting period, stratified by screening round. Increasing BMI was associated with decreased likelihood of returning to breast screening (OR = 0.993, 95% CI: 0.988–0.998; OR = 0.989, 95% CI: 0.984–0.994; OR = 0.985, 95% CI: 0.982–0.987 for women screening for the first, second and third+ time, respectively).

**Conclusions:**

This large, prospective study supports implementation of routine height and weight collection within breast screening programs. It shows that asking women their height and weight does not deter them from returning to screening and that women with increased BMI are less likely to rescreen, highlighting a need for targeted interventions to improve screening barriers for women living with obesity.

## Graphical abstract

The crude and age‐standardised rescreening rates for women 50‐72 years screened at BreastScreen Western Australia stratified by screening round, body mass index and the effect of the COVID‐19 pandemic.

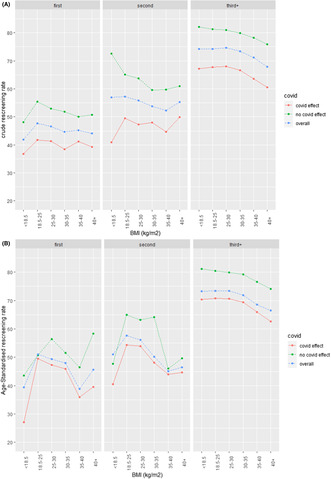

## 1 INTRODUCTION

Australia has one of the highest rates of obesity in the world, particularly in older women.^1^ More than one third of women aged 50–74 years are living with obesity (body mass index (BMI) >30 kg/m^2^) and rates are increasing.^2^ Participation in breast screening is particularly vital for women living with obesity since they are at increased risk of breast cancer,^3^ may develop more aggressive cancer types, and have poorer rates of breast cancer survival.^4^, ^5^ Women with obesity are at high‐risk of non‐participation in breast screening^6^, ^7^, ^8^, ^9^ and in Australia, women with obesity are 8% less likely to participate than women with a normal BMI.^10^ Increased BMI may also reduce the likelihood of returning to screening when next due (henceforth, rescreening), but this association is largely understudied as breast cancer screening programs do not routinely measure height and weight information.

Breast cancer screening is free for all women in Australia aged 40 years or older via the state‐run, population‐based BreastScreen programs and actively targets women between the ages 50–74 years. BreastScreen Western Australia (WA) screens ~125,000 women each year and collects core screening data at the time of each screen. In 2016, BreastScreen WA assessed the feasibility of measuring height and weight at the time of mammography by installing height and weight measuring stations in each of the mammography rooms across WA. Uptake was high, with 77% of clients agreeing to height and weight measurements over a 2‐year period.^11^ In 2018, BreastScreen WA made the decision to collect self‐reported height and weight at the time of mammography, enabling prospectively investigations of BMI associations with rescreening rates and other critical breast cancer screening outcomes.

In this study, we assess whether asking for height and weight information at the time of mammography impacts the likelihood of clients returning to screening when next due. We also use the height and weight data to investigate, for the first time, whether BMI is associated with rescreening rates within a large population‐based breast cancer screening program.

## 2 METHODS

For each screening event, core data was extracted from the BreastScreen WA database including date of birth, residential address, whether an interpreter is required for further contact, whether English is spoken at home, country of birth, Aboriginal or Torres Strait Islander status, personal and family history of breast/ovarian cancer and current hormone replacement therapy use. Screening date, screening round, screening location, whether a longer appointment was required, and whether women were recalled for further assessment was also extracted. All data was de‐identified prior to analysis.

Between 25 February 2016 and 31 January 2018, all individuals attending BreastScreen WA were eligible to have their height and weight measured by BreastScreen staff as part of their routine screening. From 1 May 2018, individuals were asked to self‐report height and weight as part of routine data collection. Height and weight were not collected for 2 months, between 1 February 2018 and 30 April 2018.

Residential postcodes were used to assign individuals to remoteness categories (major city, inner regional, outer regional, remote and very remote) using the Accessibility and Remoteness Index of Australia (ARIA)^12^ and socio‐economic status (low: decile 1–4, medium: decile 5–6 and high: decile 7–10) using deciles from index of socio‐economic disadvantage of the Socio‐Economic Indexes for Areas (SEIFA)^13^ from the Australian Bureau of Statistics 2016 census data. Screening round was recorded as the total number of screening events an individual had undergone at BreastScreen WA and categorised into three groups: first round, second round and third and subsequent rounds. Screening locations were categorised into clinic locations and mobile clinic locations. BMI was calculated using height and weight measurements and grouped into clinical categories: <18.5 kg/m^2^ (underweight), 18.5–2.5 kg/m^2^ (normal weight), 25–30 kg/m^2^ (overweight), 30–34 kg/m^2^ (obese Class I) and > 35 kg/m^2^ (obese Class II/III).

### 2.1 Definition of rescreening

The primary outcome was rescreening status, defined as whether an individual returned to screening when next due. Individuals recommended biennial screening were classified as having rescreened if they returned to BreastScreen WA within 27 months of their previous screening event and 15 months for individuals recommended for annual screening. Crude rescreening rates were calculated as the number of individuals who returned to screening within their 27‐ or 15‐month window, out of the number of individuals who had the opportunity to rescreen. Age‐standardised rescreening rates were calculated by estimating the crude rescreening rate for 5‐year age ranges, then multiplying by the age‐specific WA female population size derived from census data published by the Australian Bureau of Statistics. These weighted crude rescreening rates were summed, divided by the total WA female population size to provide the age‐standardised rate, and then multiplied by 100 so that it is expressed as a percentage.

### 2.2 Data exclusions

**FIGURE 1:**
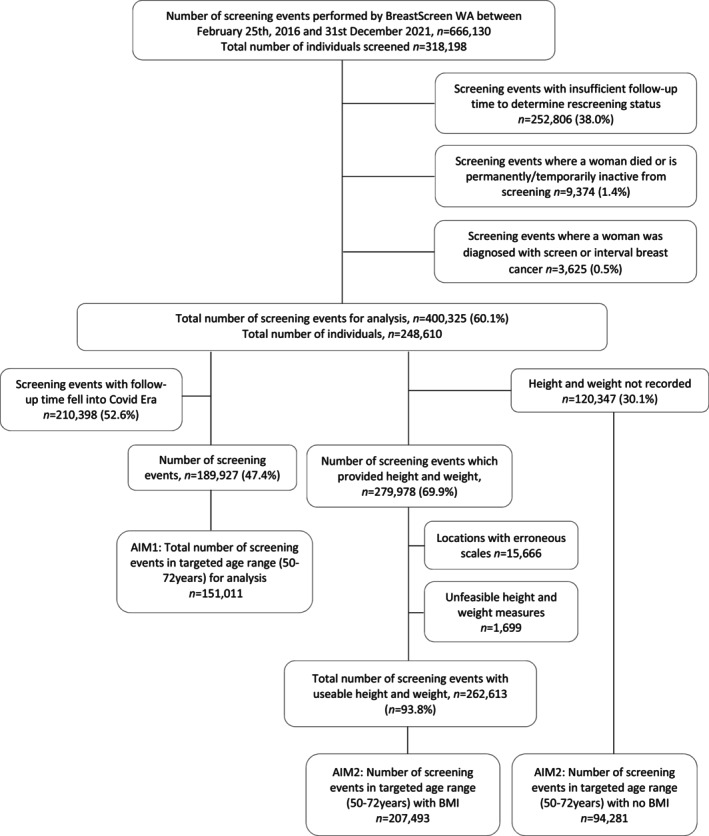
Flowchart of exclusion criteria.

Only women in the targeted age range (50–74 years) were included in this study. The data exclusion procedure is outlined in Figure 1 and describes three different groups of screening events to address two different research questions (labelled Aim 1 and Aim 2). Screening information from February 2016–December 2021 was extracted from the BreastScreen WA database (*N* = 666,130 screens from *n* = 318,198 women). Overall, screening events were excluded if an individual's rescreening status could not be determined due to insufficient follow‐up time, or if the individual became permanently or temporarily inactive from screening within the 15‐ or 27‐month follow‐up period, or if the individual was subsequently diagnosed as a screen‐ or interval‐detected breast cancer case.

BreastScreen WA closed between 30 March and 28 April, 2020 due to COVID‐19 restrictions and subsequent appointment bookings were delayed post closure. For investigations examining whether asking height and weight affects rescreening rates (henceforth, Aim 1), all screening events where the follow‐up period was scheduled post 1st January, 2020 were also excluded.

For investigations examining the association between BMI and the likelihood of rescreening (henceforth, Aim 2), erroneous height and weight measurements due to faulty scales were excluded as previously reported.^11^ The location of the faulty scales were random and unlikely to systematically bias the results. Screening events with improbable measurements of height, weight and BMI were also excluded (<100 cm and >200 cm, <25 kg and >220 kg and >70 kg/m^2^ respectively). Finally, the variation in repeated measurements over time within individuals were assessed and those whose weight measurements that had a standard deviation greater than 30 kg were also excluded for this aim.

### 2.3 Statistical analysis

The experimental unit was a screening event. Descriptive statistics were calculated separately for screening events included in Aim 1 versus Aim 2. For Aim 1, we compared crude and age‐standardised rescreening rates to those reported by the Australian Institute of Health and Welfare (AIHW) for 2014–2015,^14^, ^15^ stratified by screening round and whether height and weight was self‐reported or measured.

The distributions of the height and weight measurements were visually compared to those from the Australian Bureau of Statistics National Health Survey data, consisting of a sample of ~21,000 people across the Australian population. For Aim 2, crude and age‐standardised rescreening rates were calculated by BMI category, overall and accounting for COVID‐19 restrictions (i.e. all screening events post 30 September 2018 for annual and 30 September 2017 for biennial screeners were considered to be affected by COVID‐19 restrictions). Line graphs were used for visualisation of rescreening rates. Multivariable models were used to determine the effect of BMI, treated as a continuous variable, on rescreening status, adjusting for covariates considered important a priori to rescreening rates and BMI. The statistical program, *R*, was used for all analyses.

## 3 RESULTS

The demographic and screening characteristics of the study population are presented in Table 1. The median age at each screening event was 60–61 years old and most individuals were Caucasian/European, resided in major cities and were generally in higher quintiles of the socio‐economic indices. Over 80% of the screening events were individuals screening for the third or subsequent time and attended screening in a fixed clinic (as opposed to a mobile mammography unit). Less than 5% of screening events required a longer appointment or an interpreter. Individuals were less likely to provide height and weight measures if they were born in a country other than Australia/New Zealand/United Kingdom/Ireland, identified as Aboriginal or Torres Strait Islander, required a longer appointment or interpreter, or did not speak English at home.

**TABLE 1:** Characteristics of mammographic screening events at BreastScreen Western Australia between February 2016–September 2018 for women in the targeted age range (50–72), who had sufficient time to return to screening, stratified by whether height and weight was recorded.

Characteristic		Aim 1 *N* = 151,011	Aim 2	
			BMI not recorded *N* = 94,281	BMI Recorded *N* = 207,493
Median age (IQR)		60 (55, 66)	61 (56, 67)	60 (55, 66)
Median BMI (IQR)		NA	NA	26.9 (23.6, 31.1)
BMI category	Underweight (<18.5 kg/m^2^)	NA	NA	2414 (1.2%)
	Normal (18.5–25 kg/m^2^)	NA	NA	72,224 (34.8%)
	Overweight (25–30 kg/m^2^)	NA	NA	69,697 (33.6%)
	Obese Class I (30–35 kg/m^2^)	NA	NA	40,161 (19.4%)
	Obese Class II (35–40 kg/m^2^)	NA	NA	15,550 (7.5%)
	Obese Class III (>40 kg/m^2^)	NA	NA	7447 (3.6%)
Aboriginal or Torres Strait Islander		1957 (1.3%)	1971 (2.1%)	1891 (0.9%)
English spoken at home	Yes	129,673 (85.9%)	75,720 (80.3%)	182,420 (87.9%)
	No	21,293 (14.1%)	18,497 (19.6%)	24,994 (12.0%)
	Unknown	45 (0.0%)	64 (<0.1%)	79 (<0.1%)
Country of birth	Australia and New Zealand	90,894 (60.2%)	54,194 (57.5%)	127,880 (61.6%)
	United Kingdom and Ireland	23,935 (15.8%)	12,991 (13.8%)	33,990 (16.4%)
	Other	36,171 (24.0%)	27,071 (28.7%)	45,587 (22.0%)
	Unknown	11 (0.0%)	25 (<0.1%)	36 (<0.1%)
Family history of breast cancer	No history	116,994 (77.5%)	73,137 (77.6%)	163,213 (78.7%)
	Some family history	20,289 (13.4%)	12,620 (13.4%)	24,943 (12.0%)
	Significant family history	13,728 (9.1%)	8524 (9.0%)	19,337 (9.3%)
Hormone Replacement Therapy use	Yes	17,107 (11.3%)	8365 (8.9%)	24,215 (11.7%)
	No	133,899 (88.7%)	85,915 (91.1%)	183,276 (88.3%)
	Unknown	<5 (<0.1%)	<5 (<0.1%)	<5 (<0.1%)
Disadvantage index	Low	38,612 (25.6%)	24,960 (26.5%)	54,040 (26.0%)
	Medium	34,921 (23.1%)	23,690 (25.1%)	48,395 (23.3%)
	High	76,665 (50.8%)	45,198 (47.9%)	103,842 (50.0%)
	Missing	813 (0.5%)	433 (0.5%)	1216 (0.6%)
Remoteness and accessibility index	Major city	117,547 (77.8%)	74,577 (79.1%)	157,563 (75.9%)
	Inner and outer regional	28,506 (18.9%)	15,984 (17.0%)	42,686 (20.6%)
	Remote and very remote	4149 (2.7%)	3288 (3.5%)	6030 (2.9%)
	Missing	809 (0.5%)	432 (0.5%)	1214 (0.6%)
Screening round	First	11,824 (7.8%)	7248 (7.7%)	17,871 (8.6%)
	Second	13,864 (9.2%)	8084 (8.6%)	18,845 (9.1%)
	Third or subsequent	125,323 (83.0%)	78,949 (83.7%)	170,777 (82.3%)
Screened at clinic/mobile	Clinic	125,300 (83.0%)	78,226 (83.0%)	170,375 (82.1%)
	Mobile	25,711 (17.0%)	16,055 (17.0%)	37,118 (17.9%)
Screening Interval	Annual	30,043 (19.9%)	19,140 (20.3%)	35,853 (17.3%)
	Biennial	120,968 (80.1%)	75,141 (79.7%)	171,640 (82.7%)
Recalled for assessment		3921 (2.6%)	2597 (2.8%)	5724 (2.8%)
Longer appointment required	Yes	3680 (2.4%)	2910 (3.1%)	4523 (2.2%)
	No	146,941 (97.3%)	90,993 (96.9%)	202,585 (97.8%)
	Missing	390 (0.3%)	378 (0.4%)	385 (0.2%)
Interpreter required	Yes	834 (0.6%)	1160 (1.2%)	616 (0.3%)
	No	150,177 (99.4%)	93,120 (98.8%)	206,872 (99.7%)
	Missing	<5 (0.1%)	<5 (<0.1%)	<5 (<0.1%)

The crude and age‐standardised rescreening rates for women whose rescreens were not affected by COVID‐19 restrictions are found in Table 2. The rescreening rates for the current study time period (2016–2018) did not markedly decline compared to those previously reported for the years immediately prior (2015). For example, the age‐standardised rescreening rates for women screening for the third and subsequent round was 79.2% in the current study compared to 79.8% in 2015. Overall, rescreening rates for women screened since 2016 were within 1.8% points from the previous reporting period, stratified by screening round. The biggest differences in rescreening rates were observed upon stratification by method of collection (measured vs. self‐reported).

**TABLE 2:** Crude and age‐standardised rescreening rates for women 50–72 yrs who attended BreastScreen Western Australia stratified by screening round as reported by the Australian Institute of Health and Welfare (AIHW) compared to the current study.

Data source	Year	First round		Second round		Third and subsequent rounds	
		Crude	ASR	Crude	ASR	Crude	ASR
Australian BreastScreen Monitoring Report*	2014	55.2%	51.9%	64.7%	62.4%	81.6%	81.6%
Australian BreastScreen Monitoring Report**	2015	53.0%	51.4%	63.0%	60.9%	80.1%	79.8%
Current Study (Aim 1, Overall)	2016–2018	52.8% (6242/11,824)	52.0%	62.6% (8676/13,864)	62.7%	80.2% (100,487/125,323)	79.2%
Measured height and weight	2016–2017	52.8% (6199/11,740)	50.8%	62.6% (8502/13,592)	57.6%	80.3% (96,564/120,290)	81.0%
Self‐reported height and weight	2018	51.2% (43/84)	51.6%	64.0% (174/272)	66.0%	77.9% (3923/5033)	76.7%

* refers to reference #14.

** refers to reference #15 however, please note the typo in reference #15. It should be the 2019 report, not the 2020.

**FIGURE 2:**
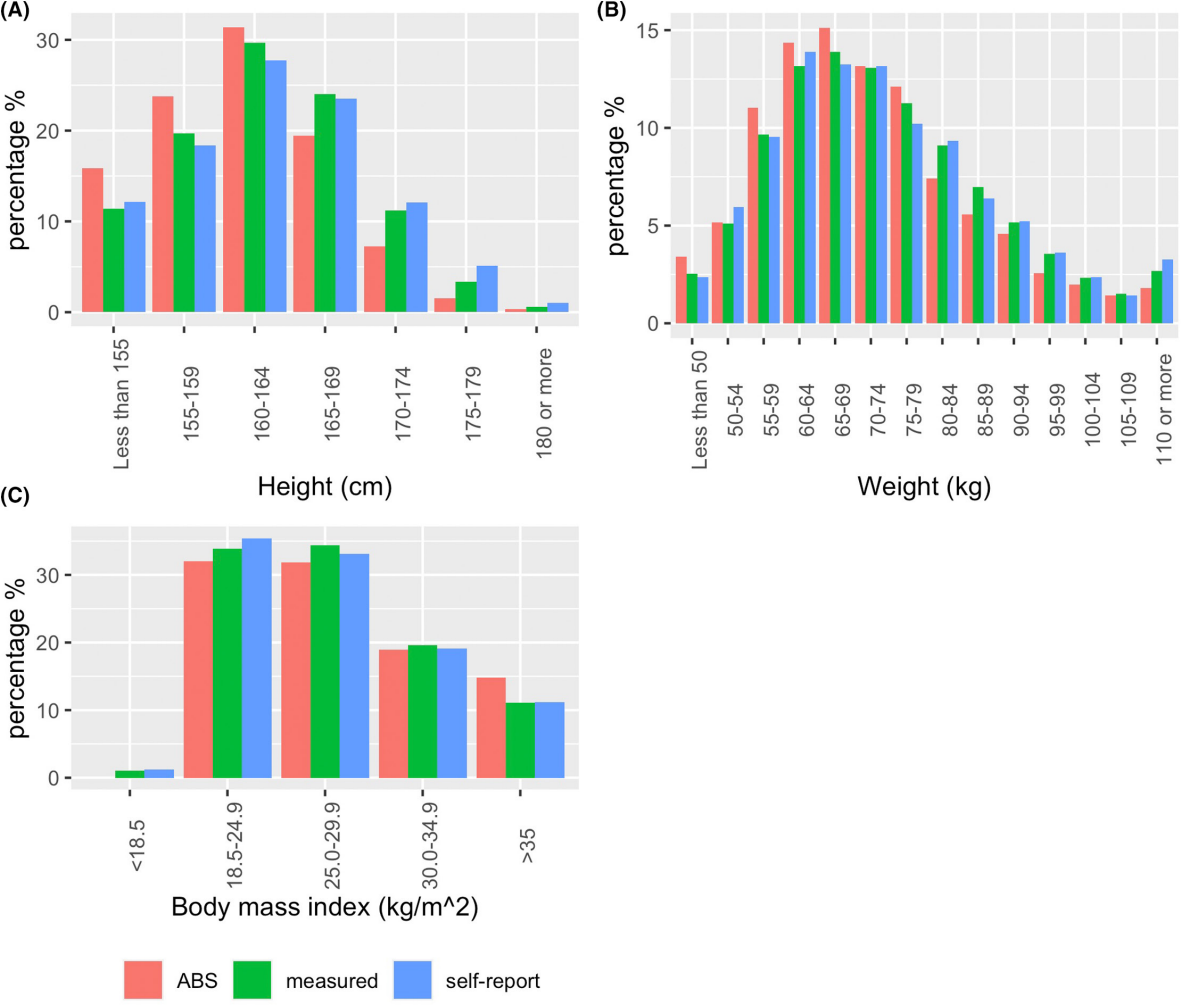
Distribution of BreastScreen Western Australia participants (A) heights, (B) weights and (C) body mass index measurements compared to Australian Bureau of Statistics National Health Study for ages 35+ years.

The height, weight and BMI distributions for our sample stratified by whether height and weight was measured or self‐reported is compared with ABS data in Figure 2. Overall, the distributions were similar, but with a higher proportion of taller and heavier women in our sample compared to that estimated by ABS for the general female population. No obvious trend was seen when comparing the height and weight distributions of those who were measured versus those who self‐reported, and the overall distribution of BMI showed little difference in measurement type by BMI category.

**FIGURE 3:**
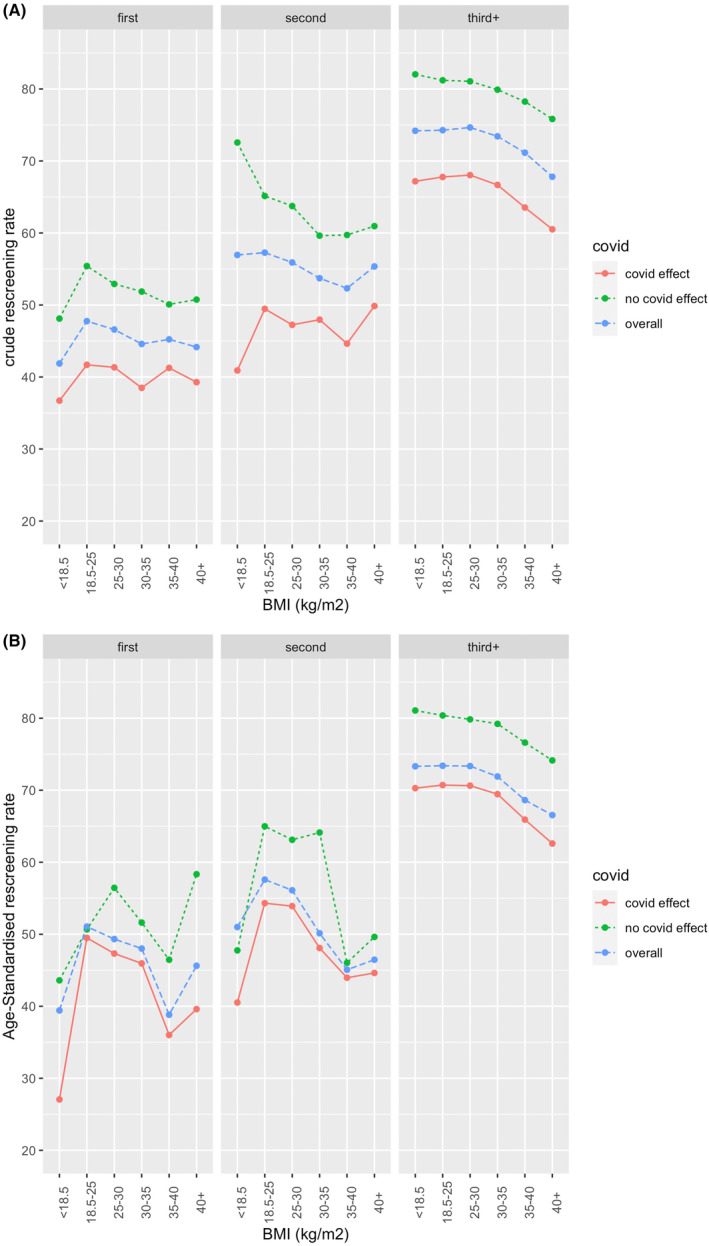
(A) Crude and (B) age standardised rescreening rates for women 50–72 yrs screened at BreastScreen Western Australia stratified by screening round, body mass index and the effect of the COVID‐19 pandemic.

Crude and age‐standardised rescreening rates by BMI category, screening round and the effect of the COVID‐19 pandemic are given in Table 3 and visualised in Figure 3. Crude rescreening rates show that as BMI increased the likelihood of rescreening decreased, particularly for those screening for their third or subsequent time. As expected, rescreening rates were higher prior to the COVID‐19 pandemic. Age‐standardised rescreening rates for third and subsequent screeners show similar trends to that of the crude rescreening rates. However, more variation was seen in the age‐standardised rescreening rates for first‐ and second‐time screeners. Multivariable adjusted odds ratios for BMI (Table 4) shows that overall, the odds of rescreening decreased as BMI increased across all screening rounds, regardless of whether screening was affected by the covid pandemic.

**TABLE 3:** Crude and age‐standardised rescreening rates for BreastScreen Western Australia stratified by screening round, body mass index (BMI) and COVID‐19 effect.

Screen round	< 18.5 kg/m^2^, *N* = 2414 ASR, % (crude)	18.5–25 kg/m^2^, *N* = 72,224 ASR, % (crude)	25–30 kg/m^2^, *N* = 69,697 ASR, % (crude)	30–35 kg/m^2^, *N* = 40,161 ASR, % (crude)	35–40 kg/m^2^, *N* = 15,550 ASR, % (crude)	≥40 kg/m^2^, *N* = 7447 ASR, % (crude)	Height and weight not recorded, *N* = 94,281 ASR, % (crude)
	Overall						
First	39.4 (98/234)	51.1 (3133/6560)	49.3 (2635/5655)	48.0 (1467/3290)	38.8 (956/2132)	45.6 (344/779)	46.7 (3223/7248)
Second	51.0 (127/223)	57.6 (3922/6848)	56.1 (3378/6042)	50.1 (1928/3589)	45.1 (1143/2143)	46.5 (398/719)	49.6 (4179/8084)
Third or subsequent	73.3 (1452/1957)	73.4 (43,686/58,816)	73.4 (43,298/58,000)	71.9 (24,439/33,282)	68.6 (13,122/18,722)	66.5 (4034/5949)	70.6 (55,843/78,949)
	No COVID effect						
First	43.6 (51/106)	50.7 (1607/2900)	56.4 (1357/2564)	51.6 (778/1500)	46.5 (305/609)	58.3 (168/331)	50.0 (1576/3103)
Second	47.8 (82/113)	65.0 (2222/3411)	63.1 (2022/3172)	64.1 (1056/1771)	46.0 (433/725)	49.6 (217/356)	62.5 (2001/3344)
Third or subsequent	81.1 (758/924)	80.4 (23,108/28,455)	79.8 (23,872/29,450)	79.2 (13,578/16,993)	76.6 (5173/6611)	74.1 (2149/,2834)	77.6 (23,387/29,928)
	COVID effect						
First	27.1 (47/128)	49.5 (1526/3660)	47.3 (1278/3091)	46.0 (689/1790)	36.0 (307/744)	39.6 (176/448)	46.1 (1647/4145)
Second	40.5 (45/110)	54.3 (1700/3437)	53.9 (1356/2870)	48.1 (872/1818)	44.0 (312/699)	44.6 (181/363)	46.8 (2178/4740)
Third or subsequent	70.3 (694/1033)	70.7 (20,578/30,361)	70.6 (19,426/28,550)	69.5 (10,861/16,289)	65.9 (3915/6162)	62.6 (1885/3115)	67.9 (32,456/49,021)

Abbreviations: ASR, Age‐Standardised Rate.

**TABLE 4:** Odd ratios and 95% Confidence interval for multivariable regression for effect of BMI on rescreening rates stratified by screening round and COVID‐19 effect.

Screen round	Multivariable adjusted odds ratio for BMI (95% CI), *N* = 205,810
Overall	
First	0.993 (0.988, 0.998)^a^ ^,^ ^b^
Second	0.989 (0.984, 0.994)^a^ ^,^ ^b^
Third or subsequent	0.985 (0.982, 0.987)^a^ ^,^ ^b^ ^,^ ^c^
No covid effect	
First^a^	0.989 (0.982, 0.997)^a^
Second^a^	0.982 (0.975, 0.990)^a^
Third or subsequent	0.987 (0.973, 1.001)^a^ ^,^ ^c^
Covid effect	
First^a^	0.996 (0.989, 1.003)^a^
Second^a^	0.996 (0.989, 1.003)^a^
Third or subsequent	0.985 (0.983, 0.988)^a^ ^,^ ^c^

^a^ Adjusted for age at screening, Aboriginal and Torres Strait Islander, country of birth, English spoken at home, hormone replacement therapy use, disadvantage index, remoteness, family history of breast cancer, recalled for assessment, clinic or mobile, screening interval, longer appointment required, and interpreter required.

^b^ Additionally adjusted for covid effect.

^c^ Multivariable analysis also included a random effect using ID.

## 4 DISCUSSION

Height and weight data are not available elsewhere in Australian public screening programs or internationally, making this large prospective database a world first. This study shows that asking women their height and weight information at the time of mammography does not deter them from returning to screening when next due, providing evidence to support the expansion of height and weight collection within/across other screening programs in Australia and internationally. This study is also likely the first to demonstrate that increased BMI is associated with decreased likelihood of returning to breast screening, suggesting a need for targeted interventions to improve screening barriers for women living with obesity.

Obesity is one of the most attributable risk factors for cancer and BMI is a strong predictor of breast cancer risk, particularly in post‐menopausal women.^3^ Increased BMI has also been shown to significantly inhibit breast cancer screening participation^6^, ^7^, ^8^, ^9^ which in Australia, prevents around 8 breast cancer deaths for every 1000 women aged between 50 and 74 years who attend biennial mammographic screening.^16^ Despite this, height and weight information is not routinely collected as part of breast cancer screening programs in Australia or internationally. Previous work has demonstrated that it is feasible to expand the core data collected by BreastScreen programs to include height and weight^11^ but for screening to be effective, women must attend routinely and screening programs need to demonstrate consistent high rescreening performance. By comparing rescreening rates to previous reporting periods, this study now demonstrates that the act of asking women their height and weight at the time of mammography did not deter them from rescreening. The method of collection also didn't appear to affect rescreening rates (recognising the limited number of screening events with self‐reported measures). Therefore, this study suggests that it is safe for other breast screening programs to collect height and weight information at the time of mammography.

To the authors' knowledge, the association between BMI and rescreening rates has not been studied before. Examination of the crude rescreening rates showed a downward trend with BMI beyond the 18–25 kg/m^2^ category for all screening rounds, but particularly for women screening for the third+ time. The impact of the COVID‐19 pandemic on reduced rescreening rates are apparent but did not appear to differ for women of differing BMI categories. Crude rescreening rates for women who did not provide height and weight information were consistent with women with a BMI >25 kg/m^2^ which is consistent with our previous finding that women who did not provide height and weight were more likely to have larger breasts.^11^ Crude rescreening rates also appear to be relatively lower for women with BMI <18 kg/m^2^ screening for the first or second time, suggesting that screening barriers related to body image are likely to exist at opposing ends of the BMI spectrum. However, the number of screening events in these sub‐groups is relatively small and therefore, the rates are not as precise as the other estimates.

Examination of the age‐adjusted rescreening rates tells a similar story to those of the crude rates but the magnitude of the downward trend between the 25–30 kg/m^2^ and the 35–40 kg/m^2^ categories was much more pronounced. The variation in the rescreening rates for women screening for the first and second time was greater compared to that for the third+ screeners, likely because there were ~10 times more screening events in the third+ group. Overall, there is a downward trend between rescreening rates and increasing BMI (excluding those with a BMI <18 kg/m^2^) but care must be taken when considering the precision of the estimated crude and age‐adjusted rates for certain sub‐groups.

The multivariable analysis addresses the issue of reduced precision of the rescreening rates in some sub‐groups by treating BMI as a continuous variable. It also enables adjustment of other potential confounders such as age, country of birth, language spoken at home, Aboriginal and Torres Strait Islander status, remoteness of living and socio‐economic status. Overall, the multivariable analysis indicated strong evidence of an association between increasing BMI and reduced likelihood of women returning to screening when next due—regardless of screening round. This suggests that targeted interventions to improve rescreening rates within women living with obesity are needed, particularly since women living with obesity are at increased risk of post‐menopausal breast cancer^3^ and have poorer rates of breast cancer survival.^4^, ^5^

Strengths of this study include its size with over 650,000 screening events from over 300,000 women. As far as the author's know, this is the only study of its kind as height and weight is not routinely collected within other population‐based screening programs in Australia or internationally. The availability of core screening data enabled high‐powered multivariable analysis, providing definitive estimates of the association between BMI and the likelihood of rescreening within a population‐based screening program. Limitations include the lack of precision of the crude and age‐adjusted rescreening rates for some of the sub‐groups. Also, around 80% of the screening events were women attending for the third/subsequent time. As BreastScreen WA continues to collect voluntary height and weight information at the time of screening, there will be more measurement data from first and second time screeners as the database matures. It will be possible to reprise the analysis at a future date, when larger numbers of women who have only screened once or twice could be reported. As it stands, the current analysis included 11,824 screening events from first‐time screeners and 13,864 from second‐round screeners and all analyses were stratified by screening round, enabling comparison between groups.

This study demonstrates one of potentially many benefits of routinely collecting height and weight information within screening programs. Co‐development of a targeted intervention to improve the mammogram experience for women in larger bodies, and thereby improve the likelihood of rescreening, is currently underway within the BreastScreen WA program. Future analyses examining the associations between BMI and other critical breast screening outcomes (e.g. recall rates, cancer detection rates and program sensitivity) is also underway. As most validated breast cancer risk prediction models require height and weight information, the addition of these key variables to existing core screening data enables more complete and accurate risk assessment on a population level.

In summary, this study provides evidence supporting the implementation of routine collection of height and weight information at the time of mammographic screening. Asking women their height and weight information at the time of screening does not appear to deter them from returning to screening when next due, a key consideration for any population‐based screening program. This study also demonstrates, for the first time, that increased BMI is associated with decreased likelihood of returning to breast screening, highlighting a need for targeted interventions to improve screening barriers for women living with obesity.

## AUTHOR CONTRIBUTIONS

**Sarah Pirikahu:** Data curation (lead); formal analysis (lead); methodology (equal); writing – original draft (lead); writing – review and editing (equal). **Ellie Darcey:** Data curation (supporting); formal analysis (supporting); methodology (supporting); writing – original draft (supporting); writing – review and editing (equal). **Helen Lund:** Conceptualization (supporting); data curation (supporting); funding acquisition (supporting); project administration (supporting); resources (supporting); writing – original draft (supporting); writing – review and editing (supporting). **Elizabeth Wylie:** Conceptualization (equal); funding acquisition (equal); investigation (supporting); project administration (supporting); resources (supporting); writing – original draft (supporting); writing – review and editing (supporting). **Jennifer Stone:** Conceptualization (lead); funding acquisition (lead); investigation (lead); methodology (equal); project administration (lead); resources (lead); supervision (lead); writing – original draft (supporting); writing – review and editing (equal).

## CONFLICT OF INTEREST STATEMENT

The authors declare no conflicts of interest.

## ETHICS STATEMENT

Ethics approval was obtained from the Western Australian Department of Health Human Research Ethics Committee (RGS0000004491).

## PRECIS

This study provides new evidence supporting the implementation of routine collection of height and weight information within population‐based screening programs. It shows, for the first time, that women with increased body mass index are less likely to rescreen, highlighting a need for targeted interventions to improve screening barriers for women living with obesity.

## CONSENT FOR PUBLICATION

All women who attend BreastScreen WA provide written consent at every screen to the collection and use of data relating to the screen, including evaluation and research purposes, provided names are not used in any reports or published statistics.

## Data Availability

The datasets generated and analysed during the current study are not publicly available but can be made available upon request (and pending approval) from BreastScreen Western Australia.
